# Association between Circulating Levels of IGF-1 and IGFBP-3 and Lung Cancer Risk: A Meta-Analysis

**DOI:** 10.1371/journal.pone.0049884

**Published:** 2012-11-19

**Authors:** Hongxin Cao, Guanghui Wang, Long Meng, Hongchang Shen, Zhen Feng, Qi Liu, Jiajun Du

**Affiliations:** 1 Institute of Oncology, Provincial Hospital Affiliated to Shandong University, Shandong University, Jinan, People’s Republic of China; 2 Department of Thoracic Surgery, Provincial Hospital Affiliated to Shandong University, Shandong University, Jinan, People’s Republic of China; University of Kentucky, United States of America

## Abstract

**Background:**

The insulin-like growth factor (IGF) system was documented to play a predominant role in neoplasia. As lung cancer is one of the most malignant cancers, we conducted a meta-analysis in order to investigate the strength of association between circulating IGF-1 and IGFBP-3 levels and lung cancer.

**Methodology/Principal Findings:**

A systematic literature search was conducted to identify all prospective case-control studies and case-control studies on circulating IGFs and IGFBPs levels. Six nested case-control studies (1 043 case subjects and 11 472 control participants) and eight case-control studies (401 case subjects and 343 control participants) were included in this meta-analysis. Pooled measure was calculated as the inverse variance-weighted mean of the natural logarithm of multivariate adjusted OR with 95% CIs for highest vs. lowest levels to assess the association of circulating IGF-1 and IGFBP-3 concentrations and lung cancer. Standard mean difference (SMD) was also calculated to indicate the difference of the circulating IGF-1 and IGFBP-3 concentrations between the lung cancer case group and the control group. Of the nested case-control studies, ORs for the highest vs. lowest levels of IGF-1 and IGFBP-3 were 1.047 (95% CI: [0.802,1.367], *P* = 0.736) and 0.960 (95%CI: [0.591,1.559], *P* = 0.868) respectively; and SMDs were −0.079 (95%CI:[ −0.169, 0.011], *P* = 0.086) and −0.097 (95%CI:[ −0.264,0.071], *P* = 0.258) for IGF-1 and IGFBP-3 respectively. As to the case-control studies, SMDs were 0.568 (95%CI:[ −0.035, 1.171], *P* = 0.065) and −0.780 (95%CI:[ −1.358, −0.201], *P* = 0.008) for IGF-1 and IGFBP-3 respectively.

**Conclusions/Significance:**

Inverse association was shown between IGFBP-3 and lung cancer in the case-control studies,and the circulating level of IGFBP-3 underwent a decline during tumorogenesis and development of lung cancer, which suggested IGFBP-3 a promising candidate for the biomarker of lung cancer.

## Introduction

The insulin-like growth factor (IGF) system is viewed as a complex multifactorial system in both physiological and pathophysiological conditions. It comprises of two ligands (IGF-1 and IGF-2), three cell-membrane receptors (insulin receptor (IR), IGF-1 receptor (IGF-1R) and IGF-2 receptor (IGF-2R)), and six high-affinity IGF binding proteins (IGFBP-1 through -6) [1]. In normal conditions, the levels of the components reach a balance, so that the IGF axis can perform as a regulator of cellular proliferation as well as cell survival. While in case the original balance is broken, it plays a predominant role in pathogenesis, of which neoplasia is currently attracting substantial interest. Sustaining proliferative signaling and evading growth suppressors which are caused by over-expression of growth factors or their receptors are now regarded as hallmarks of cancer [2].

The IGFs and IGFBPs are mainly synthesized in liver, meanwhile, they also functionate in autocrine and paracrine modes. IGF-1 has been documented to perform strong mitogenic and anti-apoptotic effects both in normal and cancerous cells [3], [4], including lung cancer cell lines [5], [6]. Most serum IGFs are not in free forms, but bonding with IGFBPs, of which IGFBP-3 is the predominant member [7]. The IGFBPs regulate the biological accessibility and activity of the IGFs by increasing the half-lives of circulating IGFs and controlling their availability for receptor binding. Beyond that, IGFBP-3 acts as an inhibitor or potentiator of IGFs independently of IGF-1 binding. In several non-small cell lung cancer (NSCLC) cell lines, IGFBP-3 acts as a potent inhibitor of IGF-1R signaling by interfering with both the MAPK and PI-3K/Akt signaling pathways, resulting in growth arrest and inducing apoptosis [8].

**Figure 1:**
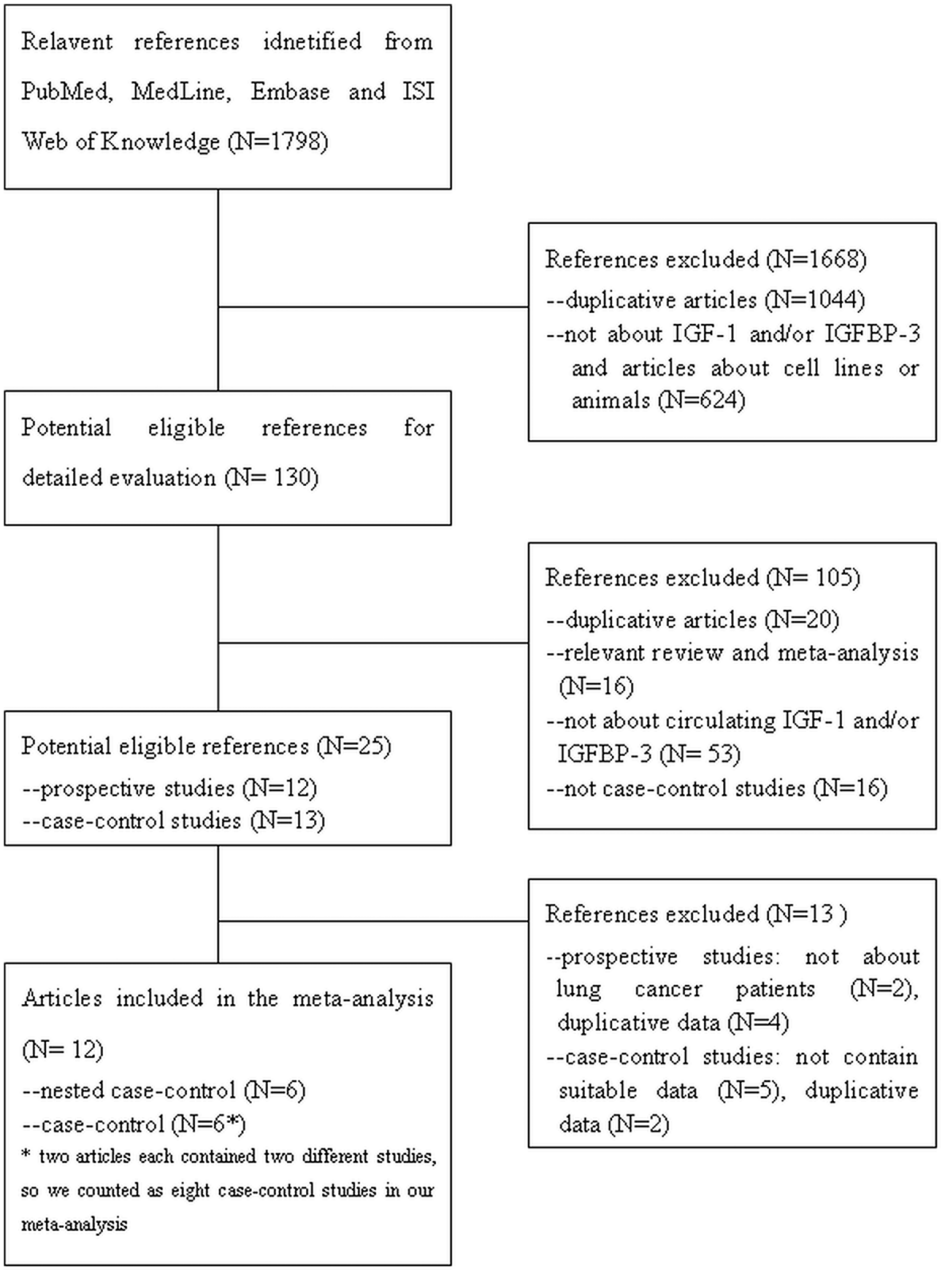
Flow diagram of included studies for this meta-analysis.

Above on, an elevated level of circulating IGF-1 and/or a lower level of IGFBP-3 was related to the increased risk of cancers, and this was supported by some studies in breast, prostate and colorectal cancers [9], [10], [11]. For risk stratification, they were supposed to be promising biomarkers of cancer for early diagnosis and prognosis. But practically, there were also some epidemiological studies showing null association between circulating IGF-1 and IGFBP-3 levels and cancers [12], [13], [14]. Also for lung cancer, several conclusions of the studies reached consistency, but even so, some appeared contradictory.

On the foundation of the inconsistent results, we performed a meta-analysis expecting to investigate the strength of association between circulating IGF-1 and IGFBP-3 levels and lung cancer.

**Table 1:** Characteristics of the nested case-control studies included in this meta-analysis.

Study	Year	Region	Age	Gender	Sample	Follow-up(years)	T(°C)	Assey method		sample size		mean(SD) for IGF-1(ng/ml)		mean(SD) for IGFBP-3 (ng/ml)		OR(95%CI)		Adjusting factors
								IGF-1	IGFBP-3	case	control	case	control	case	control	IGF-1	IGFBP-3	
Lukanova A [15]	2001	USA	32–72	F	serum	8–14	–	RIA	RIA	93	186	129.8(10.4)	131.1(7.75)	4387(166)	4413(125.5)	0.54(0.14–.07)	0.9(0.28–2.85)	time since last meal, cotinine, BMI, IGFBP-3 for IGF-1, and IGF-1 for IGFBP-3
London SJ [16]	2002	China	45–64	M	serum	8–11	-20	RIA	IRMA	230	740	123(46.43)	127(41.64)	1793(487.47)	1863(485.77)	0.86(0.47–.57)	0.5(0.25–1.02)	smoking, IGFBP-3 for IGF-1, and IGF-1 for IGFBP-3
Spitz MR [17]	2002	USA	50–69	M+F	serum	>11	−80	ELISA	ELISA	159	297	158(56)	153(54)	30700(8200)	29400(7900)	0.64(0.31–.33)	2.35(1.13–4.92)	BMI, smoking, exposure population, IGFBP-3 for IGF-1, and IGF-1 for IGFBP-3
Wakai K. [18]	2002	Japan	40–79	M+F	serum	8	−80	IRMA	IRMA	194	9351	–	–	–	–	1.74(1.08–.81)	0.67(0.45–1.01)	area, gender, age, smoking habits, BMI, and IGFBP-3 for IGF-1
Ahn J [19]	2006	Finland	50–69	M	serum	>5	–	ELISA	ELISA	200	400	137.2(52.3)	145.5(52)	2228(650)	2369(640)	0.76(0.39–.49)	0.71(0.35–1.47)	age, intervention arm, BMI, years of smoking, IGFBP-3 for IGF-1, and IGF-1 for IGFBP-3
Morris JK [20]	2006	UK	35–64	M	serum	15	−40	ELISA	ELISA	167	498	–	–	–	–	1.21(0.62–.35)	1.7(0.87–3.3)	age by matching,smoking

M,male; F,female; T, storage temperature; RIA, radioimmunoassay assay; IRMA, immunoradiometric assay; ELISA, enzyme-linked immunoabsorbent assay; BMI,body mass index.

**Table 2:** Characteristics of the case-control studies included in this meta-analysis.

Study	Year	Area	Mean age(case/control)	Gender	Cases	Sample	T (°C)	Assey method		Sample size		Mean(SD) for IGF-1(ng/ml)		mean(SD) for IGFBP-3(ng/ml)	
								IGF-1	IGFBP-3	case	control	case	control	case	control
Bhatavdekar JM [21]	1994	India	–	M	–	serum	−70	RIA	–	9	25	211.68 (219 )	33.33(41.6)	–	–
Bhatavdekar JM [21]	1994	India	–	M	–	serum	−70	RIA	–	28	25	134.11(127.31)	33.33(41.6)	–	–
Lee DY [22]	1999	Korea	-	M+F	N+S	serum	−70	RIA	WLB	41	20	207.9 (62.9)	281.3(53.9)	–	–
Wu XF [23]	2000	USA	62/63	M+F	N+S	plasma	−80	ELISA	ELISA	183	227	166(69.02)	143(61.50)	3674(931.76)	3745(937.81)
Wang H [24]	2004	China	62.5/59	M+F	N+S	serum	−20	RIA	IRMA	78	14	570.67(185.80)	427.66(141.49)	3133.60(1110.30)	4024.67(1373.31)
Unsal E [25]	2005	Turkey	60/40	M+F	N+S	serum	−40	IRMA	IRMA	24	12	126.9(63.4)	167.6(56.5)	2277.6(614.0)	2874.7(861.9)
Izycki T [26]	2006	Poland	59.4/−	M+F	N	serum	–	ELISA	ELISA	25	10	123.6(43.3)	74.2(12.0)	1385.5(344.8)	1732.4(312.1)
Izycki T [26]	2006	Poland	60.2/−	M+F	S	serum	–	ELISA	ELISA	13	10	134.5(44.8)	72.3(18.0)	1276.2(264.8)	1732.4(312.2)

M,male; F,female; N,NSCLC; S,SCLC; T, storage temperature; RIA, radioimmunoassay assay; IRMA, immunoradiometric assay; ELISA, enzyme-linked immunoabsorbent assay; WLB, Western ligand blot.

## Methods

### Data Collection and Selection Criteria for Meta-analysis

A literature search was carried out on PubMed, MedLine, Embase and ISI Web of Knowledge using the terms: “insulin-like growth factor-1” “insulin-like growth factor bonding protein-3” “lung cancer” “serum” with all possible combinations. All potentially eligible studies were retrieved, and their bibliographies were checked for other pertinent articles. Review articles and bibliographies of other pertinent articles identified were manually inspected to find additional eligible studies. The inclusion criteria in the meta-analysis were as follows: 1) prospective cohort studies, nested case-control studies and case-control studies published before August 2012; 2) articles contained data on circulating IGF-1, IGFBP-3, and lung cancer risk; 3) the most informative article when multiple articles were published by the same authors or groups. The following articles were excluded: 1) review articles without original data; 2) articles lacking data or containing data inappropriate for meta-analysis; 3) case reports and 4) overlapping articles or duplicate data. All potentially relevant articles were reviewed by two investigators independently, and the final decision was made depending on correspondence of the investigators.

**Figure 2:**
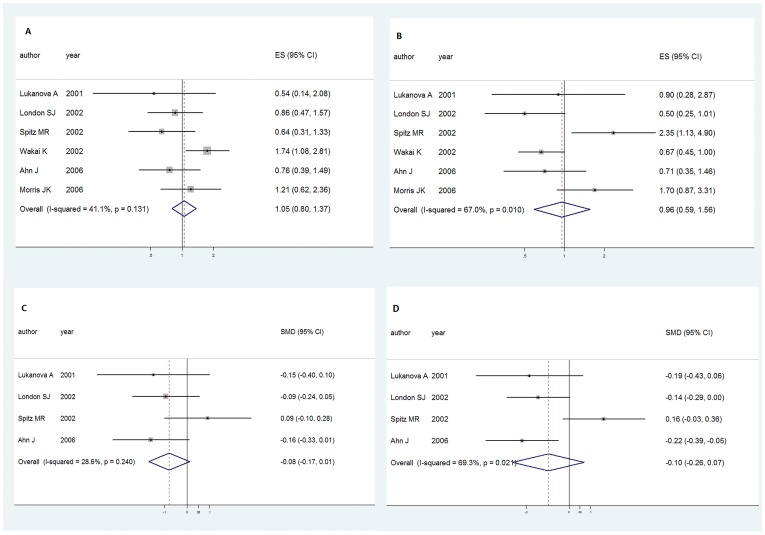
Forest plot of cancer risk associated with the IGF-1 and IGFBP-3 in nested case-control studies. A. Odds ratios with corresponding 95% CIs of the circulating IGF-1 level of individual studies and pooled data of the nested case-control studies. B. Odds ratios with corresponding 95% CIs of the circulating IGFBP-3 level of individual studies and pooled data of the nested case-control studies. C. Standard mean differences with corresponding 95% CIs of the circulating IGF-1 level of individual studies and pooled data of the nested case-control studies. D. Standard mean differences with corresponding 95% CIs of the circulating IGFBP-3 level of individual studies and pooled data of the nested case-control studies.

**Figure 3:**
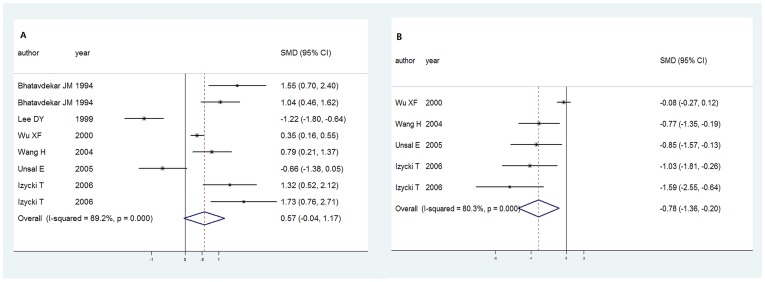
Forest plot of cancer risk associated with the IGF-1 and IGFBP-3 in case-control studies. A. Standard mean differences with corresponding 95% CIs of the circulating IGF-1 level of individual studies and pooled data of the case-control studies. B. Standard mean differences with corresponding 95% CIs of the circulating IGFBP-3 level of individual studies and pooled data of the case-control studies.

### Data Extraction

Data extraction and quality assessment were conducted independently by two investigators using a standardized protocol and data recording form. And information was examined and adjudicated independently after being extracted and assessed.

A total of 1 798 articles were retrieved after the first search in PubMed, MedLine, Embase and ISI Web of Knowledge. As shown in **Fig. 1**, six nested case-control studies [15], [16], [17], [18], [19], [20](**Table 1**)and eight case-control studies [21], [22], [23], [24], [25], [26] (two articles contained two studies that matched our needs respectively) (**Table 2**) met the criteria described in the previous section. The following data were collected from each study: name of the first author, year of publication, years of follow-up, region of the studies, mean age, gender of the cases, pathological type, assey methods of IGF-1 and IGFBP-3, number of the case and control groups, mean and standard deviation (SD) of IGF-1 and IGFBP-3, odds ratio (OR) for highest vs. lowest levels and its corresponding 95% confidence interval (CI), adjusting factors as well as other details described in the articles.

### Statistical Analyses

Definitely, a nested case-control study is comprised of subjects sampled from an assembled epidemiological prospective cohort study in which the sampling depends on disease status, that is, it compares exposures in case patients (patients in the cohort who develop disease) and a sample of individuals in the cohort who have not developed disease; case-control study is a retrospective study in which patients who already have a certain condition are compared to people who do not [27], [28], [29]. In other words, the blood samples of the nested case-control studies were collected at the beginning of the studies, that is, the IGF-1 and IGFBP-3 concentrations indicated the state of the cases several years before the detectable neoplasm appeared; whereas blood samples of the case-control studies were collected after the cases were definitely diagnosed as lung cancer patients. As a result, we hypothesized that the internal environments of the cases were of different conditions at the two stages when the blood samples were collected, so we processed the data separately in order to find any difference if it did exist.

With regard to the nested case-control studies, we used adjusted OR with 95% CIs for highest vs. lowest levels as the principal effective measure. Pooled measure was calculated as the inverse variance-weighted mean of the natural logarithm of multivariate adjusted OR with 95% CIs for highest vs. lowest levels to assess the association of circulating IGF-1 and IGFBP-3 concentrations and lung cancer. Standard mean difference (SMD) was also calculated to indicate the difference of the circulating IGF-1 and IGFBP-3 concentrations between the lung cancer case group and the control group.

For the case-control studies, we only adopted means and SDs as the applied measure to assess the circulating IGF-1 and IGFBP-3 concentrations between the lung cancer case group and the control group because ORs were not provided in most of the studies.

Heterogeneity between trials was evaluated by I-squared (*I^2^*) statistic [30]. These indices assess the percentage of variability across studies attributable to heterogeneity rather than chance. Statistical heterogeneity was considered significant when *I^2^*>50%. An *I^2^* value<50% for the *I^2^* statistic indicates a lack of heterogeneity among studies, so the pooled OR estimate of the each study was calculated by the fixed effects model (the Mantel-Haenszel method) [31]. Or else, the random-effects model (the DerSimonian and Laird method) was used [32].

The ‘leave one out’ sensitive analysis [33] was carried out using *I^2^*>50% as the criteria to evaluate the key studies with substantial impact on between-study heterogeneity. Publication bias was estimated using Egger’s regression asymmetry test [34]. An analysis of influence was conducted [35], which described how robust the pooled estimator was to removal of individual studies. An individual study was suspected of excessive influence, if the point estimate of its omitted analysis lied outside the 95% CI of the combined analysis. All reported *P* values were two-sided with significance set at<0.05. Statistical analyses were carried out using STATA 11.0 (Stata Corporation, Collage Station, Texas, USA).

## Results

### Nested Case-control Studies

Six nested case-control studies were ultimately chosen in this meta-analysis. All studies combined, a total number of 1 043 case subjects and 11 472 control participants were included. Four articles offered the means and SDs of the circulating concentrations of IGF-1 and IGFBP-3, and amount to 682 case subjects and 1 623 control participants were included.

With respect to the circulating IGF-1 concentration and lung cancer risk, initial meta-analysis has shown that it was at no significantly increased risk with the OR of 1.047(95% CI:[0.802,1.367], *P* = 0.736) for the highest vs. lowest levels of IGF-1 (**Fig. 2A**). SMD of the four articles didn’t show statistical difference between the case and control group with SMD of −0.079 (95%CI:[ −0.169,0.011], *P* = 0.086) (**Fig. 2C**).

Concerning the relationship of circulating IGFBP-3 concentration and lung cancer risk, the pooled OR 0.960 (95%CI:[0.591,1.559], *P* = 0.868) (**Fig. 2B**) as well as to SMD −0.097 (95%CI:[ −0.264,0.071], *P* = 0.258) (**Fig. 2D**) were calculated similar to that of IGF-1. The statistical power was not strong enough to demonstrate the relationship between IGFBP-3 concentration and lung cancer risk.

### Case-control Studies

Eight case-control studies were involved in this meta-analysis, among which eight included means and SDs of the circulating concentration of IGF-1, that is, a total of 401 case subjects and 343 control participants were included. The circulating concentration of IGFBP-3 was mentioned in five studies, that is, 323 case subjects and 273 control participants were involved.

In order to investigate the circulating IGF-1 concentration and lung cancer risk, the SMD 0.568 (95%CI: [-0.035, 1.171], *P* = 0.065) (**Fig. 3A**) was calculated with the means and SDs supplied in the articles. Statistically, no difference between the case and control group was seen.

As to the relationship between the circulating IGFBP-3 concentration and lung cancer risk, we also calculated the SMD −0.780 (95%CI: [−1.358, −0.201], *P* = 0.008) (**Fig. 3B**) with the means and SDs supplied in the articles. We could see inverse relationship between the circulating concentration of IGFBP-3 and lung cancer risk.

### IGF-1 and IGFBP-3 in Nested Case-control Studies and Case-control Studies

In both the nested case-control studies and case-control studies, the pooled results showed no statistical difference between the case subjects and the control participants for the circulating levels of IGF-1, indicating the circulating levels of the IGF-1 were both in the normal range at the corresponding time. When it came to the circulating IGFBP-3 levels, no difference was shown between the case subjects and the control participants in the nested case-control studies, but that of lung cancer patients’ was significantly lower than the control participants’ in the case-control studies. Circulating IGFBP-3 status detected years before the diagnosis of lung cancer was within the normal range; however, the circulating IGFBP-3 status of the confirmed lung cancer patients was lower than normal range. Evident fall of the circulating IGFBP-3 levels of the lung cancer patients was shown even though we couldn’t define the pattern and duration of the decline.

### Sensitive Analysis and Publication Bias Analysis

The ‘leave one out’ sensitive analysis was conducted using *I^2^*>50% as the criteria to evaluate the key studies with substantial impact on between-study heterogeneity. The pooled ORs and SMDs were not materially altered after the sensitive analysis.

In the Egger’s regression asymmetry test, there was no evidence of publication bias of the IGF-1 and IGFBP-3 in the nested case-control studies and IGF-1 in the case-control studies. But IGFBP-3 in the case-control studies was tested to have publication bias.

## Discussion

The pooled results of the meta-analysis didn’t show evidence of the relationship between the circulating concentrations of IGF-1 and IGFBP-3 and lung cancer risk in the nested case-control studies. The result was in accordance with most of the nested case-control studies included in this meta-analysis. Though London, S. J. et al. declared that subjects with higher serum levels of IGFBP-3 were at reduced risk of lung cancer from a prospective study of men in China [16], the pooled data didn’t show telltale of the function of high circulating IGFBP-3 level to reduced risk of lung cancer. It indicated that neither the circulating level of IGF-1 nor that of IGFBP-3 could act as long-term(the follow-up period were all more than five years and some even more than twenty years in the prospective studies we included in this meta-analysis)predictor of lung cancer.

Similarly, the meta-analysis showed no association between the circulating IGF-1 level and lung cancer in the case-control studies though the mean value of circulating concentration of IGF-1 of case subjects was higher than that of the control group in several studies involved in the meta-analysis [24], [26], [36]. But the circulating concentration of IGFBP-3 was showed to inversely associate with lung cancer. In another word, the lung cancer patients were statistically demonstrated to have lower levels of circulating IGFBP-3 compared with control participants, which suggested IGFBP-3 a promising candidate for the biomarkers of lung cancer.

What’s more,the difference between the nested case-control studies and the case-control studies highlighted our notice. The circulating IGFBP-3 concentrations of blood samples collected years before detectable lung cancer were of no difference with that of the control participants (people who involved in the prospective studies without lung cancer until the endpoint of the studies); whereas, when it came to the case-control studies, the circulating IGFBP-3 concentrations of blood samples from the definitely diagnosed lung cancer patients were significantly lower than that of the control participants. Though the results were gained from different populations, we speculated that the IGFBP-3 level underwent a decline during the process of tumorogenesis and development and remarkable fall of the circulating IGFBP-3 level might be detected during the rapidly progressing period of the tumorogenesis. This result echoed the hypothesis we mentioned before to a certain degree. We also assumed that there would be a conceivable time point that the concentration of circulating IGFBP-3 could participant in helping us to distinguish the status of people into high lung cancer risk and low lung cancer risk groups. Further research should be conducted emphasizing larger studies, pooled analyses, analyses by cancer subtype, improved exposure assessment, better and standard design categories, and possible mechanisms to corroborate the assumption.

The potential insufficiency of this meta-analysis was that the studies designs and the assey methods of serum IGF-1 and IGFBP-3 as well as other risk factors were not standardized. The nonstandard assey methods were the main cause of the heterogeneity between studies. So we used OR with 95% CIs for highest vs. lowest levels with the most adjusting factors as the principal effective measure if the original articles supplied in order to offset the insufficiency as possible as we could. Moreover, in order to identify whether the long-term storage of the blood samples would influence the concentration of IGFs, Morris, J. K. et al. carried out an experiment to test the concentrations of IGFs both the samples collected prior to 1982 which stored at −40°C and samples collected in 2003. Thereinto, the median levels of the blood samples prior to 1982 compared with the median of blood samples in 2003 were 1% (95% CI: [−12%,+25%]) higher for IGF-1 (*P* = 0.66), demonstrating that the long-term storage (over 20 years) of the serum at −40°C did not change the levels of IGF-1 [20]. So we considered that the concentrations of IGF-1 and IGFBP-3 of the serum with long-term storage were believable.

Circulating IGF-1 and IGFBP-3 absorbed the point of view of many scientists for their great potential. Efforts have been made expecting to prove the clinical significance of circulating IGF-1 and IGFBP-3 in cancers by evidence-based methods [37], [38], [39], [40], [41], [42], among which lung cancer is a magnificent being [43], [44]. With precise statistical methods and more studies included in, we got convincing results that the circulating IGFBP-3 level was inversely associated with lung cancer in the case-control studies. Practically, measurements from case-control studies might reflect tumor marker status rather than true risk assessment. It provided hopely base to translate the laboratory indicator into clinical setting as a tumor marker. Moreover, a decline of IGFBP-3 during tumorogenesis was inferred through our results.

## References

[1] Samani AA, Yakar S, LeRoith D, Brodt P (2007) The role of the IGF system in cancer growth and metastasis: overview and recent insights. Endocrine reviews 28: 20–47. 16931767 10.1210/er.2006-0001

[2] Hanahan D, Weinberg RA (2011) Hallmarks of cancer: the next generation. Cell 144: 646–674. 21376230 10.1016/j.cell.2011.02.013

[3] Pollak MN, Schernhammer ES, Hankinson SE (2004) Insulin-like growth factors and neoplasia. Nat Rev Cancer 4: 505–518. 15229476 10.1038/nrc1387

[4] Khandwala HM, McCutcheon IE, Flyvbjerg A, Friend KE (2000) The effects of insulin-like growth factors on tumorigenesis and neoplastic growth. Endocr Rev 21: 215–244. 10857553 10.1210/edrv.21.3.0399

[5] Macaulay VM, Everard MJ, Derrick Teale J, Trott PA, Van Wyk JJ, et al (1990) Autocrine function for insulin-like growth factor I in human small cell lung cancer cell lines and fresh tumor cells. Cancer research 50: 2511. 2156621

[6] Quinn KA, Treston AM, Unsworth EJ, Miller MJ, Vos M, et al (1996) Insulin-like growth factor expression in human cancer cell lines. Journal of Biological Chemistry 271: 11477–11483. 8626706 10.1074/jbc.271.19.11477

[7] Kelley KM, Oh Y, Gargosky SE, Gucev Z, Matsumoto T, et al (1996) Insulin-like growth factor-binding proteins (IGFBPs) and their regulatory dynamics. Int J Biochem Cell Biol 28: 619–637. 8673727 10.1016/1357-2725(96)00005-2

[8] Lee HY, Chun KH, Liu B, Wiehle SA, Cristiano RJ, et al (2002) Insulin-like growth factor binding protein-3 inhibits the growth of non-small cell lung cancer. Cancer Res 62: 3530–3537. 12068000

[9] Schairer C, McCarty CA, Isaacs C, Sue LY, Pollak MN, et al (2010) Circulating insulin-like growth factor (IGF)-I and IGF binding protein (IGFBP)-3 levels and postmenopausal breast cancer risk in the prostate, lung, colorectal, and ovarian cancer screening trial (PLCO) cohort. Horm Cancer 1: 100–111. 21761353 10.1007/s12672-010-0013-y PMC10358053

[10] Stattin P, Rinaldi S, Biessy C, Stenman UH, Hallmans G, et al (2004) High levels of circulating insulin-like growth factor-I increase prostate cancer risk: a prospective study in a population-based nonscreened cohort. J Clin Oncol 22: 3104–3112. 15284261 10.1200/JCO.2004.10.105

[11] Vatten LJ, Holly JM, Gunnell D, Tretli S (2008) Nested case-control study of the association of circulating levels of serum insulin-like growth factor I and insulin-like growth factor binding protein 3 with breast cancer in young women in Norway. Cancer Epidemiol Biomarkers Prev 17: 2097–2100. 18708402 10.1158/1055-9965.EPI-08-0212

[12] Schernhammer ES, Holly JM, Hunter DJ, Pollak MN, Hankinson SE (2006) Insulin-like growth factor-I, its binding proteins (IGFBP-1 and IGFBP-3), and growth hormone and breast cancer risk in The Nurses Health Study II. Endocr Relat Cancer 13: 583–592. 16728584 10.1677/erc.1.01149

[13] Roddam AW, Allen NE, Appleby P, Key TJ (2008) Endogenous sex hormones and prostate cancer: a collaborative analysis of 18 prospective studies. J Natl Cancer Inst 100: 170–183. 18230794 10.1093/jnci/djm323 PMC6126902

[14] Otani T, Iwasaki M, Sasazuki S, Inoue M, Tsugane S (2007) Plasma C-peptide, insulin-like growth factor-I, insulin-like growth factor binding proteins and risk of colorectal cancer in a nested case-control study: the Japan public health center-based prospective study. Int J Cancer 120: 2007–2012. 17266031 10.1002/ijc.22556

[15] Lukanova A, Toniolo P, Akhmedkhanov A, Biessy C, Haley NJ, et al (2001) A prospective study of insulin-like growth factor-I, IGF-binding proteins-1, -2 and -3 and lung cancer risk in women. Int J Cancer 92: 888–892. 11351312 10.1002/ijc.1265

[16] London SJ, Yuan JM, Travlos GS, Gao YT, Wilson RE, et al (2002) Insulin-like growth factor I, IGF-binding protein 3, and lung cancer risk in a prospective study of men in China. J Natl Cancer Inst 94: 749–754. 12011225 10.1093/jnci/94.10.749

[17] Spitz MR, Barnett MJ, Goodman GE, Thornquist MD, Wu X, et al (2002) Serum insulin-like growth factor (IGF) and IGF-binding protein levels and risk of lung cancer: a case-control study nested in the beta-Carotene and Retinol Efficacy Trial Cohort. Cancer Epidemiol Biomarkers Prev 11: 1413–1418. 12433720

[18] Wakai K, Ito Y, Suzuki K, Tamakoshi A, Seki N, et al (2002) Serum insulin-like growth factors, insulin-like growth factor-binding protein-3, and risk of lung cancer death: a case-control study nested in the Japan Collaborative Cohort (JACC) Study. Jpn J Cancer Res 93: 1279–1286. 12495466 10.1111/j.1349-7006.2002.tb01235.x PMC5926930

[19] Ahn J, Weinstein SJ, Snyder K, Pollak MN, Virtamo J, et al (2006) No association between serum insulin-like growth factor (IGF)-I, IGF-binding protein-3, and lung cancer risk. Cancer Epidemiol Biomarkers Prev 15: 2010–2012. 17035415 10.1158/1055-9965.EPI-06-0580

[20] Morris JK, George LM, Wu T, Wald NJ (2006) Insulin-like growth factors and cancer: no role in screening. Evidence from the BUPA study and meta-analysis of prospective epidemiological studies. Br J Cancer 95: 112–117. 16804529 10.1038/sj.bjc.6603200 PMC2360494

[21] Bhatavdekar JM, Patel DD, Chikhlikar PR, Mehta RH, Vora HH, et al (1994) Levels of circulating peptide and steroid hormones in men with lung cancer. Neoplasma 41: 101–103. 8208311

[22] Lee DY, Kim SJ, Lee YC (1999) Serum insulin-like growth factor (IGF)-I and IGF-binding proteins in lung cancer patients. Journal of Korean Medical Science 14: 401–404. 10485619 10.3346/jkms.1999.14.4.401 PMC3054398

[23] Wu XF, Yu H, Amos CI, Hong WK, Spitz MR (2000) Joint effect of insulin-like growth factors and mutagen sensitivity in lung cancer risk. Journal of the National Cancer Institute 92: 737–743. 10793110 10.1093/jnci/92.9.737

[24] Wang H, Wan YX, Zhang QK (2004) [Significance and expression of insulin-like growth factor 1 and IGF binding protein 3 in serum of patients with lung cancer]. Ai Zheng 23: 710–714. 15191678

[25] Unsal E, Koksal D, Yurdakul AS, Atikcan S, Cinaz P (2005) Analysis of insulin like growth factor 1 and insulin like growth factor binding protein 3 levels in bronchoalveolar lavage fluid and serum of patients with lung cancer. Respiratory Medicine 99: 559–565. 15823452 10.1016/j.rmed.2004.10.012

[26] Izycki T, Chyczewska E, Naumnik W, Ossolinska M (2006) Serum levels of IGF-I and IGFBP-3 in patients with lung cancer during chemotherapy. Oncol Res 16: 49–54. 16783968 10.3727/000000006783981251

[27] Langholz B (2005) Case–Control Study, Nested. Encyclopedia of Biostatistics.

[28] Nahler G (2009) nested case-control studies. Dictionary of Pharmaceutical Medicine: 120–120.

[29] Ernster VL (1994) Nested case-control studies. Preventive Medicine 23: 587–590. 7845919 10.1006/pmed.1994.1093

[30] Higgins JP, Thompson SG, Deeks JJ, Altman DG (2003) Measuring inconsistency in meta-analyses. BMJ 327: 557–560. 12958120 10.1136/bmj.327.7414.557 PMC192859

[31] Mantel N, Haenszel W (1959) Statistical aspects of the analysis of data from retrospective studies of disease. J Natl Cancer Inst 22: 719–748. 13655060

[32] DerSimonian R, Laird N (1986) Meta-analysis in clinical trials. Control Clin Trials 7: 177–188. 3802833 10.1016/0197-2456(86)90046-2

[33] Patsopoulos NA, Evangelou E, Ioannidis JP (2008) Sensitivity of between-study heterogeneity in meta-analysis: proposed metrics and empirical evaluation. Int J Epidemiol 37: 1148–1157. 18424475 10.1093/ije/dyn065 PMC6281381

[34] Egger M, Davey Smith G, Schneider M, Minder C (1997) Bias in meta-analysis detected by a simple, graphical test. BMJ 315: 629–634. 9310563 10.1136/bmj.315.7109.629 PMC2127453

[35] A T (1999) Assessing the influence of a single study in the meta-analysis estimate. Stata Tech Bull 47: 15–17.

[36] Wu X, Yu H, Amos CI, Hong WK, Spitz MR (2000) Joint effect of insulin-like growth factors and mutagen sensitivity in lung cancer risk. J Natl Cancer Inst 92: 737–743. 10793110 10.1093/jnci/92.9.737

[37] Shi R, Berkel HJ, Yu H (2001) Insulin-like growth factor-I and prostate cancer: a meta-analysis. Br J Cancer 85: 991–996. 11592771 10.1054/bjoc.2001.1961 PMC2375097

[38] Rowlands MA, Gunnell D, Harris R, Vatten LJ, Holly JM, et al (2009) Circulating insulin-like growth factor peptides and prostate cancer risk: a systematic review and meta-analysis. Int J Cancer 124: 2416–2429. 19142965 10.1002/ijc.24202 PMC2743036

[39] Sugumar A, Liu YC, Xia Q, Koh YS, Matsuo K (2004) Insulin-like growth factor (IGF)-I and IGF-binding protein 3 and the risk of premenopausal breast cancer: a meta-analysis of literature. Int J Cancer 111: 293–297. 15197785 10.1002/ijc.20253

[40] Key TJ, Appleby PN, Reeves GK, Roddam AW (2010) Insulin-like growth factor 1 (IGF1), IGF binding protein 3 (IGFBP3), and breast cancer risk: pooled individual data analysis of 17 prospective studies. Lancet Oncol 11: 530–542. 20472501 10.1016/S1470-2045(10)70095-4 PMC3113287

[41] Duan QH, Wang ZG, Zhu GB, Lu ZX, Shi LY, et al (2005) [Study on the relations between serum insulin-like growth factor-1, insulin-like growth factor binding protein-3 and colorectal cancer: a meta-analysis]. Zhonghua Liu Xing Bing Xue Za Zhi 26: 132–134. 15921617

[42] Rinaldi S, Cleveland R, Norat T, Biessy C, Rohrmann S, et al (2010) Serum levels of IGF-I, IGFBP-3 and colorectal cancer risk: results from the EPIC cohort, plus a meta-analysis of prospective studies. Int J Cancer 126: 1702–1715. 19810099 10.1002/ijc.24927

[43] Renehan AG, Zwahlen M, Minder C, O’Dwyer ST, Shalet SM, et al (2004) Insulin-like growth factor (IGF)-I, IGF binding protein-3, and cancer risk: systematic review and meta-regression analysis. Lancet 363: 1346–1353. 15110491 10.1016/S0140-6736(04)16044-3

[44] Chen B, Liu S, Xu W, Wang X, Zhao W, et al (2009) IGF-I and IGFBP-3 and the risk of lung cancer: a meta-analysis based on nested case-control studies. J Exp Clin Cancer Res 28: 89. 19549343 10.1186/1756-9966-28-89 PMC2706806

